# DNA Methylation Clusters and Their Relation to Cytogenetic Features in Pediatric AML

**DOI:** 10.3390/cancers12103024

**Published:** 2020-10-17

**Authors:** Jatinder K. Lamba, Xueyuan Cao, Susana Raimondi, James Downing, Raul Ribeiro, Tanja A. Gruber, Jeffrey Rubnitz, Stanley Pounds

**Affiliations:** 1 Department of Pharmacotherapy and Translational Research, College of Pharmacy, University of Florida, Gainesville, FL 32608, USA; 2 Department of Acute and Tertiary Care, University of Tennessee Health Science Center, Memphis, TN 38163, USA; xcao12@uthsc.edu; 3 Department of Pathology, St. Jude Children’s Research Hospital, Memphis, TN 38105, USA; Susana.Raimondi@STJUDE.ORG (S.R.); James.Downing@STJUDE.ORG (J.D.); 4 Department of Oncology, St. Jude Children’s Research Hospital, Memphis, TN 38105, USA; Raul.Ribeiro@STJUDE.ORG (R.R.); Jeffrey.Rubnitz@STJUDE.ORG (J.R.); 5 Department of Pediatrics, Stanford University School of Medicine, Stanford, CA 94305, USA; tagruber@stanford.edu; 6 Department of Biostatistics, St. Jude Children’s Research Hospital, Memphis, TN 38105, USA; Stanley.Pounds@STJUDE.ORG

**Keywords:** acute myeloid leukemia, DNA methylation, cytogenetics, pediatrics

## Abstract

**Simple Summary:**

Acute myeloid leukemia is a heterogenous disease with dismal outcome. In order to improve currently used therapeutic strategies it is important to get a in depth understanding of the molecular and genomic landscape of AML. Adult AML studies have established the significant of mutational profile of epigenetic genes as well as epigenetic deregulation DNA methylation signatures. In the current study we focused on establishing the DNA methylation profile in pediatric AML. Our result show that in pediatric AML patients the risk group and cytogenetic features have distinctive epigenetic signatures. Additionally, we observed that distinctive epigenetic hotspots co-occur complementary to the known genomic lesions and contribute towards leukemogenesis.

**Abstract:**

Acute Myeloid Leukemia (AML) is characterized by recurrent genetic and cytogenetic lesions that are utilized for risk stratification and for making treatment decisions. In recent years, methylation dysregulation has been extensively studied and associated with risk groups and prognosis in adult AML, however, such studies in pediatric AML are limited. Moreover, the mutations in epigenetic genes such as *DNMT3A*, *IDH1* or *IDH2* are almost absent or rare in pediatric patients as compared to their abundance in adult AML. In the current study, we evaluated methylation patterns that occur with or independent of the well-defined cytogenetic features in pediatric AML patients enrolled on multi-site AML02 clinical trial (NCT00136084). Our results demonstrate that unlike adult AML, cytosine DNA methylation does not result in significant unique clusters in pediatric AML, however, DNA methylation signatures correlated significantly with the most common and recurrent cytogenetic features. Paired evaluation of DNA methylation and expression identified genes and pathways of biological relevance that hold promise for novel therapeutic strategies. Our results further demonstrate that epigenetic signatures occur complimentary to the well-established chromosomal/mutational landscape, implying that dysregulation of oncogenes or tumor suppressors might be leveraging both genetic and epigenetic mechanisms to impact biological pathways critical for leukemogenesis.

## 1. Introduction

Acute Myeloid Leukemia (AML) is the second most common childhood leukemia and despite advances over the past decade it accounts for significant mortality among childhood cancer patients. AML is a heterogeneous disease characterized by recurrent genetic lesions that contribute to disease pathogenesis and also serve as important predictors of outcome in AML patients. These features are thus used for classification of patients into different risk groups with the low-risk groups AML patients primarily characterized by presence of t(8;21)/*RUNX1-RUNX1T1*, t(15;17)/*PML-RARA*, inv(16)/*CBFB-MYH11*, *NPM1* and *CEBPA* mutations; and high-risk group AML characterized by presence of features such as inv(3)/*GATA2/EV1*, t(5;11)/*NUP98-NSD1*, del(5q), monosomy 7, complex karyotype, *ASXL*, *FLT3-ITD*, *MLL-PTD* and *RUNX1* mutations. Patients lacking any of the low or high-risk features are routinely classified as intermediate or standard-risk group [1,2]. The new ELN2017 risk classification guideline takes into consideration the recurrent cytogenetic abnormalities and genetic mutations as well as FLT3-ITD allelic ratios [3].

In addition to the genetic lesions, deregulation of epigenomic machinery contributes significantly to the pathogenesis of AML. Genome-wide DNA methylation profiling in adult AML has identified specific patterns of methylation that correlate with genomic lesions [4,5,6,7,8,9,10]. Of the sixteen differentially methylated clusters identified in adult AML, eleven correlated with known cytogenetic features with the additional signatures of potential for use in further refining the risk classification [8]. Though DNA methylation has been studied extensively in adult AML, there is limited evaluation of its diagnostic and prognostic impact in childhood AML. On top of that, recurrent mutations in several epigenetic genes such as *DNMT3A*, *TET2*, *IDH1*, *IDH2*, *EZH2* [1,11] with significant roles in pathogenesis as well as clinical outcomes have been established in adult AML, however these mutations in epigenetic regulators such as *DNMT3A* and *IDH2* are rare in pediatric AML [12,13,14]. Comparison of promoter methylation of limited number of genes (*n* = 5 genes) *P15*, *CDH1*, *ER*, *MDR1* and *RARB2* in context of *NPM1*, *CEBPA*, *FLT3* and *WT1* mutations between childhood and adult AML demonstrated age-related differences in frequency of mutations as well as in methylation patterns [15]. A recent comprehensive genomic characterization of pediatric AML from Children’s Oncology group showed that though similar to adults, pediatric AML also has low rate of overall somatic mutation burden, and the mutational profile is significantly different. Unlike adult AML, DNMT3A mutations and mutations in TP53 were almost absent and mutations in *IDH1* or *IDH2* were rare in pediatric patients [16]. These observations indicate that understanding the epigenomic and genomic landscape of pediatric AML and further its topology with transcriptomics/genomics will be critical for our understanding of AML landscape and for development of novel treatment strategies. Thus, the objective of the current study was to evaluate the genome-wide methylation and expression patterns in the context of well-defined cytogenetic and morphological features in pediatric AML patients treated on the multi-site AML02 clinical trial (NCT00136084). 

## 2. Materials and Methods

### 2.1. Patient Population

The patient cohort consisted of de novo pediatric and adolescent AML patients (<22 years of age) enrolled on St. Jude AML02, a multi-center clinical trial (ClinicalTrials.gov, number NCT00136084). Of the 232 patients enrolled in AML02 trial, 175 patients with high-quality methylome data generated from diagnostic leukemic cells were included in this study. Details of the patient characteristics, risk group classification as well study outcome have been reported previously [17]. Briefly, low-risk group were classified as having t(8;21)/*AML1-ETO[RUNX1T1-RUNX1]*, inv(16)/*CBF*β-*MHY11*, or t(9;11)/*MLL-AF9[KMT2A-MLLT3]*; high-risk group were those with monosomy 7, *FLT3*-ITD, t(6;9), megakaryoblastic leukemia, treatment-related AML, or AML arising from myelodysplastic syndrome; and the rest of the patients were provisionally classified as having standard-risk AML. Patients were randomized to induction I therapy with either high (3 g/m^2^ every 12 h on day 1, 3, and 5) or low dose (100 mg/m^2^ every 12 h on days 1–10) of cytarabine along with daunorubicin and etoposide. Bone marrow was examined on day 22 to evaluate treatment response measured as minimal residual disease (MRD22). All the samples included in the study had consent for use of specimens for research purposes either from patients, parents or guardians as well as assent from the patients as appropriate.

### 2.2. DNA Methylation Profiling

Details of DNA methylation profiling of specimens from diagnostic leukemic cells is described elsewhere [18]. Briefly, bone marrow aspirates were obtained at diagnosis, and mononuclear cells isolated using Ficoll-Hypaque density gradient centrifugation were used for isolating genomic DNA. Majority of samples had >80% leukemic cells, and for samples <80%, blasts samples were enriched by using magnetic cell sorting. Bisulfite-converted DNA (using Zymo EZ DNA Methylation kit; Zymo Research) was hybridized to Illumina Infinium 450K methylation Beadchips at University of Minnesota Genomics Center. The Hybridization fluorescent signals were read using an Illumina Beat Station GX scanner. All samples achieved greater than 95% call rate. The data was controlled for batch effects and QC performed as described previously [18]. SWAN method [19], as implemented in the R package minfi [20], was used to obtain normalized M-values for the Illumina 450K methylation array data. An ANOVA model was fit to these normalized M-values to adjust for batch effects. The batch-adjusted normalized M-values were used in subsequent statistical analysis. 

### 2.3. Transcriptomic Profiling

Genome-wide gene expression data were generated using U133A gene chips on RNA samples from diagnostic leukemic blasts as described previously [21]. Details on RNA isolation, labeling and scanning of Affymetrix arrays have been published elsewhere [21]. Expression data were normalized by the Affymetrix MAS5 algorithm and log2-transformed prior to subsequent statistical analysis.

### 2.4. Statistical Analysis

A bootstrap procedure [22,23] was used to evaluate the reproducibility and distinctiveness of methylation and transcription subgroups defined by 4032 hierarchical clustering methods (HCMs) that used one of eight criteria (standard deviation, MAD score, Hartigan’s dip statistic for multimodality [24], the most information spacings test, the sum of squared least median squares t-statistics, Sarle’s bimodality coefficient [25], the Shapiro-Wilk test of normality [26] and a weighted univariate Dunn Index statistic) to select m = 1, 2, 3, …, 9, 10, 20, 30, …, 90, 100, 200, 300, …, or 1000 probe-sets (28 possibilities) to define k = 2, 3, 4, …, 9, or 10 subgroups (9 possibilities) with average or complete linkage (2 possibilities, 8 × 28 × 9 × 2 = 4032 total HCMs). The mean proportion of subjects assigned to a subgroup, the mean proportion of times the observed assignment was reproduced, and the mean of the Dunn Index [27] across the bootstraps were respectively computed as metrics of assignment probability, reproducibility, and distinctiveness of the subgroups defined by an HCM. For each number of subgroups (k = 2, 3, …, 10), the HCM with the greatest product of these metrics was chosen as a candidate HCM for further consideration. This gave nine candidate HCMs that were then evaluated in greater detail; the empirical results of the bootstrap procedure and the association with cytogenetics tested by chi-squared test were considered in choosing the HCM. Supplementary Materials provide a more detailed description of this analysis.

## 3. Results

Infinium HumanMethylation 450K bead chip was used to obtain genome-wide methylation profiles for 175 pediatric AML patients; of these, 151 patients also had gene-expression data from Affymetrix 133A platform available. Patient characteristics are summarized in Table S1. 

### 3.1. Bootstrap Clustering for Discovery of Methylation and Transcription Subgroups

**Figure 1:**
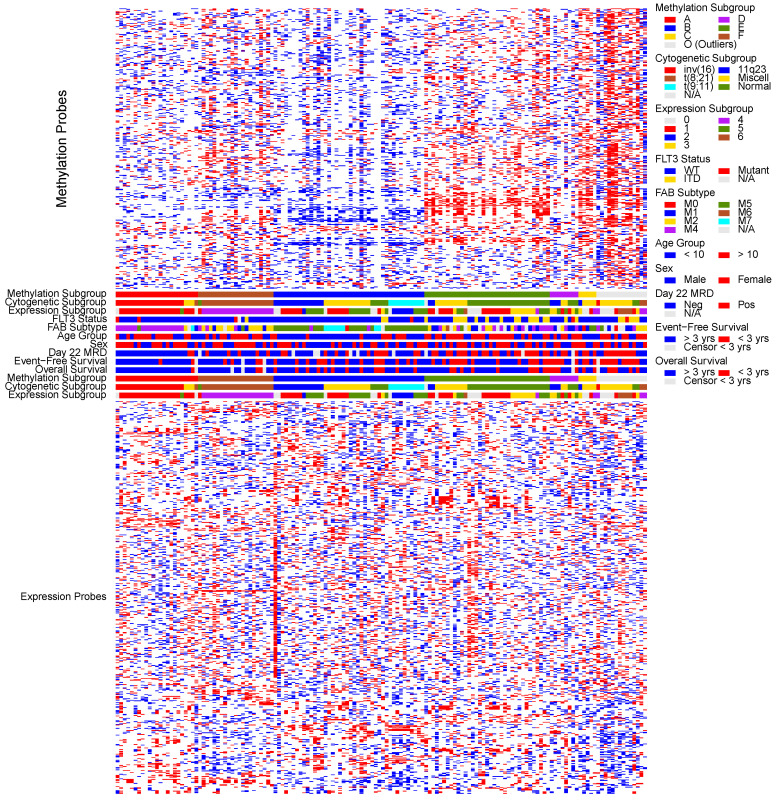
Heatmap representing top results for the unsupervised hierarchical clustering of methylation (500 probes) and expression (700 probes) probes representing different clusters across AML patients from AML02 clinical trial. For methylation, red depicts hypermethylation and blue corresponds to hypomethylation; for expression heat map at the bottom of the figure, red represents high expression and blue represents low expression. Cytogenetic features (as per color code on right), FLT3 status (FLT3-WT = blue; FLT3-ITD = yellow, FLT3-Mutation = red, FLT3-status not available = grey), FAB subtype (as per code on right); age group (<10 years = blue, >10 years = red), sex (male = blue, female = red); Day 22 MRD after induction 1 (negative = blue, positive = red, not available = grey), 3 year event free and 3 year overall survival are shown corresponding to the key on the right. For all the features, grey = data not available or censored <3 year for survival endpoints.

We used a bootstrap procedure to evaluate the subgroup assignment proportion, reproducibility, and distinctiveness of 4032 hierarchical clustering methods (HCMs) applied to our methylation and transcriptomic profiles. For each analysis and each number of subgroups considered (k = 2, 3, 4, …, 10), we identified the HCM with the best product of these empirical criteria. We then examined the subgroup assignments of these HCMs and noted that some HCMs successfully identified known cytogenetic subgroups that were missed by HCMs with slightly better empirical bootstrap criteria values. We used the empirical bootstrap criteria values and the association with cytogenetics to choose the methylation and expression HCM results to report in Figure 1. 

### 3.2. Unsupervised Discovery of Methylation Subgroups

**Figure 2:**
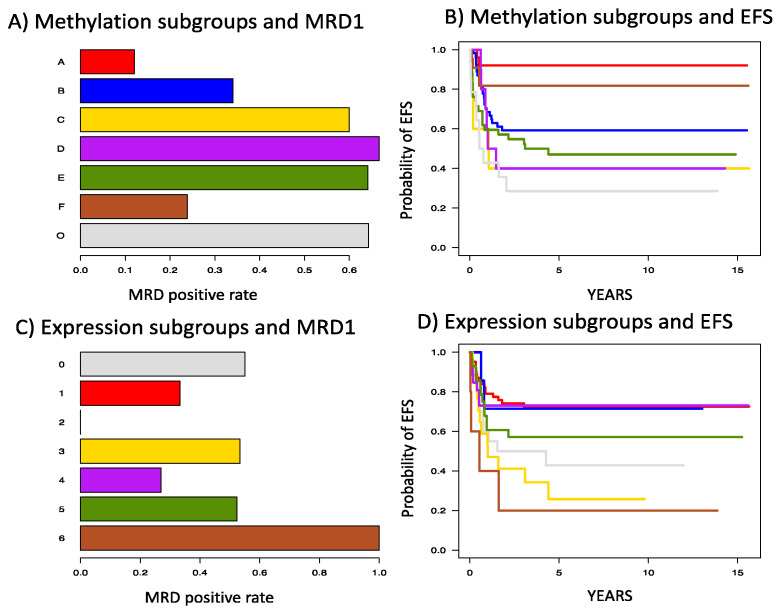
Association of the methylation clusters with (**A**) minimal residual disease after induction 1 (MRD1) and (**B**) Event free survival (EFS) and expression clusters with (**C**) MRD1 and (**D**) EFS in AML patients treated on AML02 clinical trial. The methylation and expression colors are as defined in Figure 1.

Methylation HCM #906 consisted of 500 probe-sets mapping to 304 genes with the greatest values of the Hartigan dip statistic for multimodality and complete linkage to assign 92% of subjects to one of 7 subgroups with 78.4% reproducibility and a mean bootstrap Dunn Index of 0.50. The methylation subgroups were closely aligned with cytogenetic subgroups (Figure 1 and Table 1). Methylation subgroups A, B, and F aligned very closely with inv(16), MLL-rearranged AML, and t(8;21), respectively. Methylation subgroup B included several patients with normal and miscellaneous karyotype. These patients share the methylation profiles similar to patients with MLL-rearrangements. Methylation clusters C, D, and E further subdivided normal and miscellaneous karyotype patients. Methylation group O (outliers) represented outliers with distribution predominantly in miscellaneous karyotype. The methylation subgroups had distinct clinical outcomes as well (Figure 2) with subgroups A–F having day 22 MRD positive rates of 12% (3/25), 34%(16/47), 60% (3/5), 67% (6/9), 64% (25/39), and 21% (5/21), respectively (Figure 2A). The outliers (subgroup O) had a day 22 MRD positive rate of 64% (9/14). The subgroups also demonstrated distinct EFS outcomes that aligned well with the MRD (Figure 2B). 

Several genes represented in these methylation clusters have shown to be of relevance to AML and included: *GSTM1* for which a methylation probe-set cg18938907 (GSTM1) had the 5th strongest evidence for bimodal methylation levels and showed markedly less methylation in subgroups A, C, E than in other subgroups, *GSTM1/GSTT1* deletions been implicated in AML and might be a contributor to the observed bimodal distribution [28,29]. *HOXA5* probe-set cg02916332 was hypermethylated in subgroup C, consistent with the previous reports showing it to be differentially methylated and expressed across molecular subgroups of AML [30,31]. *RPRM* is a putative AML tumor suppressor for pediatric AML [32] and the methylation probe-set cg25925441 showed hypermethylation in subgroup F relative to other subgroups. *NSD1*, that partners with *NUP98-NSD1* fusion in AML has been associated with induction failure [33], and in our cohort the *NSD1* methylation probe-set cg23383189 demonstrated distinctive hypermethylation in subgroups C and E relative to other subgroups. Some other genes identified in this analysis that are linked to AML in the literature include *PP1R13L*, *DCC*, *FOXO3*, *HOXD13*, *APP*, *MIR193A*, *HIST2HBF*, *NGFR*, *EGFL7*, *PIK3R1*, several members of proto-cadherin gene family (*PCDH*), *RFC3*, *TFAP2A*, and *DGKZ*. The complete list of methylation probe-sets identified in this analysis is available in Table S2.

### 3.3. Unsupervised Discovery of Expression Subgroups

Expression HCM 2463 used the 700 probe-sets with greatest median absolute deviation to assign 88.9% of subjects to 7 subgroups by average linkage clustering with 74.1% reproducibility and a mean bootstrap Dunn Index of 0.65. One subgroup included only 3 subjects and was combined with the other 17 outliers. The expression subgroups also demonstrated statistically significant association with cytogenetic subgroups (*p* < 0.001; Table 1; Figure 1), but this association was not as distinct as was observed with the methylation subgroups. Expression subgroup 4 aligned very closely with the t(8;21) cytogenetic subgroup, but other expression subgroups were not aligned closely with one specific cytogenetic subgroup. Nevertheless, the expression subgroups showed great variability in both MRD and EFS (Figure 2C,D).

The unsupervised analysis of gene expression also identified several genes of known relevance to AML including the fusion partners *RUNX1* and *RUNX1T1* of the common t(8;21) translocation, the commonly mutated *WT1* gene, and *MPO*, that is routinely characterized at diagnosis. Not surprisingly, *RUNX1T1* (205528_s_at) is overexpressed in expression subgroup 4 which aligns closely with the t(8;21) cytogenetic subtype. *RUNX1* (211182_x_at) and *WT1* (206067_s_at) were overexpressed in expression subgroup 3. *MPO* (203948_s_at), that codes for myeloperoxidase, a hallmark of myeloid lineage was under-expressed in expression subgroup 2. *HOXA9* (209905_at) had the greatest median absolute deviation in expression and was highly expressed in subgroups 2, 3, and 5 in comparison to the other subgroups. Subgroups 2, 3, and 5 include many MLL-rearranged cases, consistent with published studies showing that MLL is essential for *NUP98-HOXA9* induced leukemia [34]. Some other AML genes identified by this analysis include *BASP1*, *AKT3*, *ADAM28*, *VCAN*, *CTSG*, *DBN1*, *DUSP1*, *ELA2*, *ALDH1A1*, *ALDH2*, *WDFY3*, *FOXO3*, *HOXA10*, *INHBA*, *ABCB1*, *PRKCA*, *PCDHA6*, *BCL2L1*, *SLC2A3*, *EVI1*, *ZFP36L2*, *TCF4*, *VEGFA*, *BAALC*, *GFI1B*, *CDCA3*, *SOCS1*, *TNFSF10*, *SOCS3*, *RRP9*, *CD9*, *CD28*, *CD34*, *CEP135*, *FAM30A*, and *CDC42* with complete list provided in Table S3.

### 3.4. Supervised Comparisons of Cytogenetic Subgroups and Methylation-Expression Pairs Unique to Cytogenetic Features

The close alignment of the epigenomic subgroups with well-known cytogenetic classifications prompted us to perform a supervised and integrated comparison of methylation and transcription across the cytogenetic subgroups. We compared the median expression and median methylation of each probe-set across six cytogenetic subgroups [t(8;21), inv(16), t(9;11), other MLL-rearrangements, normal karyotype, and miscellaneous abnormalities]. For each subgroup, we ranked features by the difference between the median of the specific subgroup and the least and greatest median for the other subgroups. We used a bootstrap procedure to quantify the reproducibility of these rankings across 100 cohorts obtained by resampling subjects with replacement (Supplementary Materials section). Table 2 summarizes top 50 methylation features associated within each subgroup; all of the top 50 methylation features that were unique to t(8;21) and t(9;11) were hypomethylated; for normal cytogenetics 56% (28/50) and for other abnormalities 94% (47/50) of the probes were hypermethylated. Moderate levels of hypermethylation were observed for inv(16), with 12% (6/50) and other 11q23 rearrangements with 28% (14/50) probes hypermethylated. 

**Figure 3:**
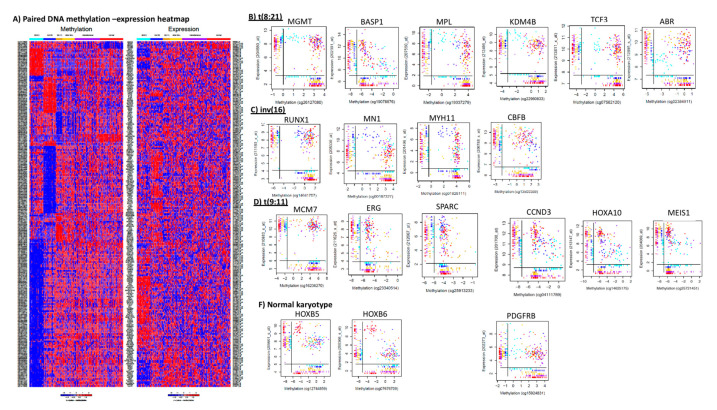
(**A**) Paired methylation and expression heatmap showing most significant features within each of the cytogenetic groups. Selected genes unique to different cytogenetic subgroups are shown in panels on right: (**B**) Inv(16); (**C**) t(8:21); (**D**) t(9:11); (**E**) other 11q23 MLL rearrangements; (**F**) normal cytogenetics and (**G**) Miscellaneous karyotype. Color for each cytogenetic group corresponds to the bar on top of the heatmaps.

Figure 3 shows the results of the paired heatmap of methylation and gene-expression signatures across different cytogenetics. Individual gene-expression methylation levels within each cytogenetic feature are included in the Table S4. Section below highlights some of the methylation/gene-expression signatures unique to each of the cytogenetic subgroups.

### 3.5. t(8;21) vs. Non-t(8;21) Comparison

Patients with t(8;21) demonstrated overall hypomethylation as compared to other groups, with all of the top 50 probes (mapping to 44 unique genes) significantly hypo-methylated as compared to non t(8;21) cases (Table 2). These genes included *MCF2L*, a guanine nucleotide exchange factor implicated in gemcitabine resistance that also impacts Rho/Rac signaling [35]; *SLC9A1*, involved in maintaining alkaline pH and Warburg effect and associated with chemo-resistance in solid tumors; *FAM120B* located in close proximity to *MLLT4*, a known fusion partner in leukemia; *DZIP1*, zinc finger protein 1, an oncogene involved in wnt/B catenin and Hedgehog signaling; genes with potential tumor suppressive effects; *PTPRF* that acts via deactivating ERK1/2 signaling; *LARP1* that interacts with oncogenic transcripts and regulates mTOR post-transcriptionally with impact on CDK9 and mTOR interaction in leukemia [36]. A few selected examples from the t(8:21) specific paired methylation and expression signatures, are shown in Figure 3 panels A and B (Table S3 shows the detailed results). For *BASP1*, that codes for a Brain acid soluble peptide, hypermethylation and corresponding low expression was seen in t(8;21) AML and a subset of miscellaneous karyotype cases. This is consistent with a recent report that methylation-associated silencing of this gene via *DNMT3A* contributes to leukemogenesis [37]. In contrast to t(8;21), we observed a reverse pattern of hypomethylation and higher expression of *BASP1* in t(9;11) and other MLL rearranged AMLs. Other genes with hypomethylation and corresponding higher expression in t(8;21) vs. other subtypes included: *MGMT* (methylguanine-DNA methyltransferase), a tumor suppressor gene that has been associated with risk of AML development [38]; *MPL* (myeloproliferative leukemia virus oncogene), which has been shown to be essential for survival and self-renewal of human preleukemic t(8;21) cells [39]. Wildtype *MPL* has been shown to be overexpressed in t(8;21) AML and promote leukemia development via *PI3K/AKT* axis activation [40]; *KDM4B*, a histone specific demethylase, has been implicated in regulating expression of genes required for maintenance of hematopoiesis [41]; *TCF3*, a fusion partner of PBX1 and 4-HLF in ALL [42,43,44,45], however its importance in AML is not currently known; and *ABR* that codes for an Active BCR related is involved in deactivation of RAC1 (ras-related C3 botulinum toxin substrate 1) important for hematopoiesis and leukemia [46]; we observed hypomethylation and low expression of *ABR* in t(8;21) (Figure 2B). Recently, *ABR* has been shown to be an enhancer of *C/EBPa*, a key mediator of myeloid differentiation, associated with azacytidine-induced apoptosis and as a favorable prognostic factor in AML [46].

### 3.6. inv(16) vs. Non inv(16)

Among the genes unique to inv(16) were: *BAG3*, a BCL2-associated gene associated with cell proliferation, chemo-resistance and antiapoptotic property with significant low expression in *NPM1* mutated vs. *NPM-WT* AML [47]; *C7orf41*, a TPA responsive gene with potential role in promotion of leukemic and normal megakaryocyte differentiation [48]; *DPF3*, a member of BAF chromatin remodeling complex with its loss linked to JAK2/STAT3 signaling pathway activation [49]; several tumor suppressers as, *CYGB*, (impacts glucose metabolism pathway); *PTPRF* with role in ERK1/2 and EGF signaling, *BANP*, a BTG3 associated nuclear protein that negatively regulates p53 expression [50]; *ARHGEF2* and *RXFP1* with role G protein coupled receptor signaling pathway; *RPTOR-* regulatory associated protein of mTOR complex which in turn has been associated with tumor growth and metastasis; *CSF1*, colony stimulating factor involved in macrophage differentiation with small molecule inhibitors being currently developed for AML [51]. Within the paired methylation and expression analysis we observed distinctive pattern for *CBFB* and *MYH11* with low-expression and hypermethylation of *CBFB* and high-expression and hypomethylation of *MYH11* (Figure 2C) in *CBFB-MYH11* fusion inv(16) cases. This indicates that genomic translocations impact the local methylation and gene expression patterns. Intriguingly, inv(16) cases exhibited significant hypomethylation (*p* = 1.13 × 10^−15^) and greater expression of *RUNX1* (also known as AML1). *RUNX1* is involved in the defining fusion of the t(8;21) cytogenetic subtype and is a member of the core-binding factor family of transcription factors, is frequently mutated and is part of chromosomal rearrangements in AML. *RUNX1-RUNX1T1* fusion in t(8;21) and *CBFB-MYH11* fusion in inv(16), are considered as driver mutations in AML. These results indicate important genomic fusions may be accompanied by epigenomic modifications at or near the affected genomic loci. These are consistent with reports indicating that RUNX1 is required for CBFB-MYH11 to induce leukemogenesis in mouse models [52]. Our results suggest that demethylation of *RUNX1* may contribute to greater expression of normal RUNX1 in pediatric *CBFB-MYH11* AML. Furthermore, consistent with other reports, we observed that *MN1* (meningioma 1) is hypomethylated and overexpressed in inv(16) AML. *MN1* overexpression is an important step in inv(16) AML leukemogenesis and its overexpression has been linked to loss of DNMT3B activity [53,54]. In murine mouse models, MN1-induced leukemia involves interactions with *MEIS1* and *HoxA9*, [55,56]; consistent with this, our results show significant inverse correlation between methylation levels and expression of *MEIS1* in inv(16), t(9;11) and other MLL patients.

### 3.7. t(9;11) vs. Non-t(9;11)

All of the top 50 probes (Table 2) with distinctive methylation in t(9;11) cases were hypo-methylated and included: *BCL2L10*, an antiapoptotic gene indicated in Myc-induced leukemogenesis [57], and a biomarker predictive of azacytidine response in MDS/AML; *MTOR*, a key player in PI3K/AKT/mTOR pathway and a pro-survival factor in leukemic stem cells and several malignancies; *IRF8*, a tumor suppressor, and cofactor of PU.1 that regulates expression levels of survival genes and has been shown to be deregulated in AML [58]; several genes of significance in ERK1/2 and PI3AKt pathways such as *RAB31* (a Ras superfamily member), C*PA4* (carboxypeptidase with role in STAT3/ERK signaling); those with role in Wnt signaling pathway including *CCDC88C*, *ANKRD6*. Within the expression-methylation paired analysis, a 27-gene signature was unique in t(9;11) subgroup. Figure 2D shows the expression-methylation correlation among different subgroups for some of t(9;11) specific genes such as: transcription regulator-*MCM7* with significant hypomethylation and overexpression in t(9;11) and some MLL-rearrangement cases; *ERG*, an ETS-related gene, was hypermethylated and under-expressed in t(9;11) (Figure 2C). *ERG* is involved in a fusion with *ELF4* and *FUS* in AML, and *ERG-FUS* rearranged AML has been shown to be associated with poor outcome in pediatric AML [59,60]. *SPARC*, a cysteine-rich acidic matrix-associated protein was hyper-methylated and under-expressed in t(9;11) and subsets of other MLL rearrangements, normal karyotype, and miscellaneous karyotype AML (Figure 2C pane3). This is consistent with low or absence of *SPARC* in AML with MLL rearrangements and its upregulation in cytogenetically normal AML with IDH2 and ERG mutations [61]. Other genes specific to t(9;11) included *MMP2*, a matrix metallopeptidase that is currently being targeted by numerous inhibitors, kinases as *AATK* (apoptosis associated tyrosine kinase), *CDK2* (cyclin dependent kinase2), *PI4KB* (Phosphoatidyl-inositol-4 kinase beta), *RPS6KA2* (ribosomal S6 kinase 2), *STK17B* (Serine threonine kinase) and *TK2* (thymidine kinase).

### 3.8. Other 11q23 MLL vs. Non-Other 11q23

Fourteen of the top 50 uniquely methylated CpGs were hypermethylated in other 11q23 rearranged AML and included genes (Table 2) such as: *EPS15*, a tyrosine kinase substrate implicated in *MLL-ALP5/EPS15* fusion in therapy-related ALL and in AML with trilineage dysplasia [62]; *KLF4*, Kruppel like factor 4 that is downregulated in *NPM1*-mutated AML. *KLF4- P53-KL4-CEBPA*-axis has also been shown to activate *CEBPA* gene transcription via p53 [63]. *HDAC1* has been shown to modulate *KLF4* expression, suggesting HDAC1 and *KLF4* as potential new molecular markers and targets for clinical diagnosis, prognosis, and treatment of myeloid leukemia [64]; *TBL1XR1* is a fusion partner of several genes in leukemias such as *TBL1XR1-RARA* fusion in APL, *TBL1XR1-ROS1* fusions in JMML, *TBL1XR1-PDGFRB*, a novel fusion in AML patients with *DEK-NUP214* fusion [65]. *TBL1XR1* is also identified as a recurrent abnormality in *ETV6-RUNX1* positive ALL. Other hypermethylated CpGs specific to other 11-q23 AML mapped to *PRMD1*, also known as Blimp1, a zinc finger transcriptional repressor; *RARRES3*, a class II tumor suppressor with role in B-CLL progression; *ZNRF2*, regulated by Akt and involved in mTOR signaling. Among significant hypomethylated targets specific to other-11q23 cases, were genes *EN1*, a member of EHG family of homeobox genes reported to be deregulated in AML [66]; *RASA3*, located at one of the aberrant regions in AML [67]; *FRMD3*, is part of 7 genes that map to commonly deleted region of chromosome 9 [del(9q)] in AML; *SPAG6*, with a role in PI3K/Akt1 pathway-mediated apoptosis and is also part of six leukemia-associated genes for measuring MRD in AML [68]. As shown in Figure 3E, *CCND3*, cyclin D3 that forms complex with CDK6 and promotes cell cycle progression was hypomethylated and overexpressed in other 11q23 MLL, t(9;11) and some normal cytogenetics AML and conversely was hypermethylated and under-expressed in t(8;21). Recurrent *CCND3* mutations have been reported in MLL-rearranged AML, and consistent with our results high *CCDN3* expression in MLL and low CCND3 expression in t(8;21) has been reported previously. *HOXA10* showed overall negative correlation between methylation and expression with patients with MLL rearrangements, t(9;11) and normal cytogenetics clustered towards lower methylation and higher expression in contrast to t(8;21). *HOXA10* is overexpressed in AML with significant dysregulation in NPM1 mutated and MLL rearranged AML, and plays a role in development to leukemia [69] by regulating expression levels of several downstream genes as *FGF2*, *TGFβ2*, *ARIH2*, *CDX4*, etc. and as a fusion partner with *NUP98*. MLL rearranged AML is characterized by overexpression of HOXA9, *HOXA10*, *MEIS*, *PBX3* and *MEF2C*. We observed similar pattern for *MEIS1*, a HOX cofactor of significant relevance in AML, suggesting differential epigenetic regulation of *HOXA10* and *MEIS1* genes as contributor to observed expression differences within cytogenetic subgroups. 

### 3.9. Other Miscellaneous Abnormalities vs. Other Cytogenetic Features

Patients with other abnormalities were enriched with significant hypermethylation of genes (with 47 of the top 50 CpGs hypermethylated; Table 2). Genes of interest with uniquely hypermethylated CpGs within this group included: *TREM2*, a tumor suppressor involved in Wnt/β catenin, ERK and PIK3/AKT/βcatenin signaling in colorectal and hepatocellular cancer; *RREB1* (RAS-responsive element-binding protein 1), a transcription factor involved in RAS signaling pathway; *KLF6* (Kruppel like transcription factor) identified as a novel mediator of *RUNX1-ETO*[*RUNX1T1-RUNX1*] [t(8;21) target gene]; *IGF2BP2*, an oncogene with negative correlation with the *CEBPA* mutation status. A recent metanalysis has shown *IGF2BP2* expression to be associated with poor prognostic factors, such as presence of *FLT3-ITD*, *IDH1* mutation and poor cytogenetic features as well as with worse overall survival in AML patients [70]; *TP53BP2*, apoptosis-stimulating protein of *TP53BP2* is a tumor suppressor that interacts with p53 family members and promotes transcriptional activation of pro-apoptosis genes; *AZU1* associated with myeloid differentiation and has been reported to be deregulated in t(8;21) AML; *CDA* (cytidine deaminase) involved in inactivation of cytarabine, backbone of AML chemotherapy; and *PDGFRB* (plate-derived growth factor receptor beta) involved in fusions with *CEV14*, *ETV6*, *CSFIR* in AML (Figure 3G). 

### 3.10. FLT3-ITD vs. Non-FLT3-ITD

FLT3 codes for FMS-like tyrosine kinase (FLT3) and mutations (specifically the most common one being an ‘internal tandem duplication-ITD’ for exons 14 and 15) and has been associated with poor prognosis AML patients. Among the genes with distinctive methylation in FLT3-ITD vs. FLT3-WT group were *CBFA2T3*, a fusion partner in *CBFA2T3-GLIS2* generated by the inv(16)(p13.3q24.3) discussed above; *ITGA2*, a *RUNX1* target gene involved in platelet aggregation and adhesion with implication in AML predisposition in familial platelet disorders (30545930) and overexpression associated with poor AML prognosis [71]. *HOXB3* was hypomethylated in *FLT3-ITD* cases, consistent with previous reports showing upregulation of *HOXB2* and *HOXB3* as novel regulators of oncogenic FLT3-ITD driven AML [72]. *STAT5A* and *TLR9*, both of which interact with *BTK*, a functionally relevant target downstream of FLT3-ITD were hypermethylation in FLT3-ITD vs. non FLT3-ITD AML cases. Table S5 lists the top 100 genes differing by FLT3-status. 

### 3.11. Complimentary Epigenetic and Genetics Hits of Significant Relevance

**Figure 4:**
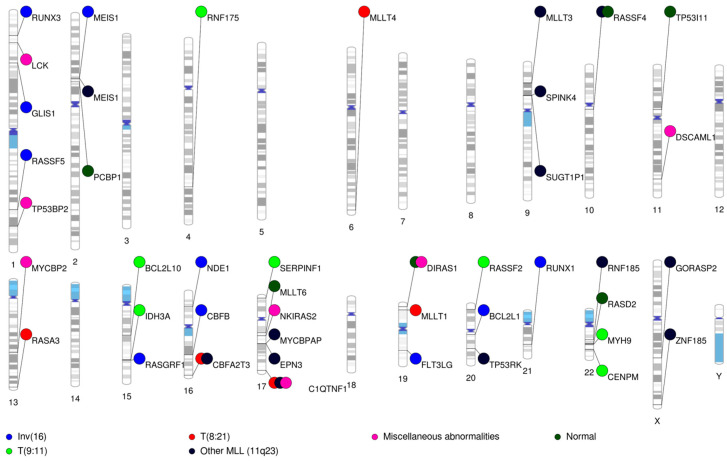
Chromosome map depicting chromosomal location of the differential methylated genes unique to the cytogenetic features. Key to the cytogenetics—blue: inv(16); red: t(8:21); pink: miscellaneous; dark green: normal cytogenetics; green: t(9:11); and black: other MLL (11q23) rearranged AML.

As has been described above within each cytogenetic group, we observed several epigenetic lesions that complimented some of the well-established cytogenetic abnormalities in AML (summarized in Table 3 and Phenogram in Figure 4). Some of the important ones include: (i) methylation of *CBFB* and *MYH11* in *CBFB-MYH11* fusion inv(16) cases indicating genomic lesion impacting local epigenetic signals; (ii) *RUNX1* and *RUNX-1T1* involved in the t(8;21) translocation, were differentially methylated in the inv(16) subgroup; (iii) within inv(16) patients, we observed hypomethylation and lower expression of *RUNX3*, where *RUNX3* regulates *RUNX1* with both having mutually exclusive expression. (iv) *CBFA2T3* demonstrated differential methylation within t(8;21) subgroup. *CBFA2T3*, is a fusion partner in *CBFA2T3-GLIS2* generated by the inv(16)(p13.3q24.3) that impacts sonic hedgehog and bone morphogenic protein pathways and is a master transcriptional co-regulator of hematopoiesis; (v) distinctive methylation of *MLLT3* and *MLLT6* in 11q23 and cytogenetically normal patients. Additionally we observed multiple genes in RAS pathway to be differentially methylated across the cytogenetic risk groups: *RASSF5* and *RASGRF1* in inv(16) cases; *RASA3* in t(8;21) cases; *RASSF2* in t(9;11); *RASSF4* in other MLL-(11q23); *DIRAS1* in miscellaneous abnormalities and normal cytogenetics AML, highlighting the significance of these pathways and genes impacted in different cytogenetic subgroups. TP53 related genes were differentially methylated within other MLL (*TP53RK*), miscellaneous (*TP53BP2*) and normal (*TP53I11*) cytogenetics groups and *BCL2L1* and *BCL2L10* were impacted in cases with inv(16) and t(9;11), respectively. This pattern of co-localization of methylation alterations in AML hotspot regions with distinctive relationships with the cytogenetic subgroups indicates altered epigenetics-mediated transcriptomic dysregulation complements the activity of the well-known fusion-genes that define cytogenetic subgroups of AML. 

### 3.12. Pathway Analysis

**Figure 5:**
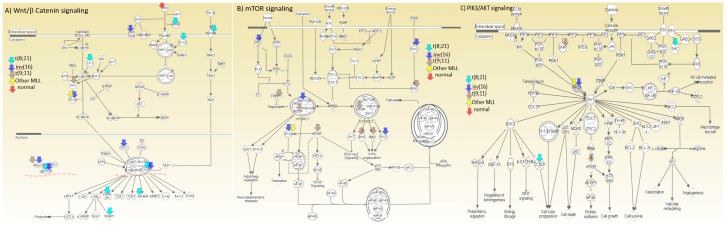
Representation of selected pathways (**A**) Wnt/b-catenin, (**B**) mTOR, and (**C**) PIK3 signaling pathways representing genes identified within different cytogenetic groups. Arrow color corresponds to the respective cytogenetic feature.

Pathway analysis using Ingenuity pathway analysis tool of top unique subtype specific methylation sites across the 6 subtype [inv(16), t(8;21), t(9;11), other (11q23) MLL-rearranged AML, normal karyotype, other abnormalities] identified specific pathways that are impacted in cytogenetics specific manner. Several genes with a role in Wnt/β catenin (*CCND1*, *CK1/2*, *Grucho*, *LRP1/5/6*, *PP2A*, *PPARδ*, *SFRP*, *SOX*, *TCF(LEF)*, *TGFR*, *AXIN1*, *Wnt*), mTOR signaling (*RAPTOR*, *PKC*, *mTOR*, *PP2A*, *RHO*, *PKC*), PIK3C/AKT signaling (*CCND1*, *SHC1*, *SHIP*, *PP2A*, *PP2A*, *mTOR*), JAk/Stat signaling (*SHC*, *MTOR*, *PI3K*, *STAT*), and Toll like receptor signaling (*IRAK*, *NIK*, *TLR9*, *TNFα*) were impacted within specific subtypes or across subtypes. Some of these are highlighted in Figure 5. 

## 4. Discussion

Dysregulated DNA methylation is a hallmark of several cancers including hematological malignancies. In this study, we used the Illumina 450K methylation array to interrogate the methylation status of more than 485,000 markers in diagnostic tumor samples from pediatric AML patients treated on the multi-site AML02 clinical trial (NCT00136084). Differentially methylated patterns that occur with or independent of the well-defined cytogenetic and morphological features in pediatric AML patients were identified and their corresponding biological roles discussed. An unsupervised analysis identified 7 methylation and 7 expression clusters, which demonstrated remarkably high degree of concordance with known cytogenetically defined subgroups. Our results from supervised analysis further confirmed methylation patterns unique to the cytogenetic subtypes and the paired methylation-expression analysis further defined cytogenetic feature-specific methylation patterns that correlated with the gene-expression signatures implying subtype-specific epigenetic regulation. 

Of interest, we report recurring complimentary epigenetic hits that impact the known mutational/ cytogenetic hotspots. Of significant relevance is methylation-mediated deregulation of *RUNX1* (a fusion partner in t(8;21) in patients with inv(16), and deregulation of *CBFA2T3*, a fusion partner in *CBFA2T3-GLIS2* generated by the inv(16)(p13.3q24.3) that impacts sonic hedgehog and bone morphogenic protein pathways in patients with (t8;21) and other MLL rearrangements. Further, multiple MLL genes show differential methylation in t(8:21) patients. In addition, we observed set of recurring methylation signatures that though contributed to subtype-specific expression regulation also altered expression regardless of subtype. These included: *CBFB* which is a fusion partner of *CBFB-MYH11* in inv(16) and demonstrated hypomethylation-dependent overexpression in other subtypes. *KMT2A* also known as MLL is located at 11q23 breakpoint and is involved in multiple translocations resulting in wide range of fusion partners in MLL-rearranged AML. KMT2A itself is a histone methyltransferase that predominantly deregulates RNA polymerase II via its fusion partners. Several of the genes that are potential targets of KMT2A were shown to be regulated by methylation in subtype-specific manner or across subtypes and included *BAHCC*, *HOXA9*, *HOXA1010*, *MEIS1*, etc. These results demonstrate that common recurring genetic alterations and subtype-specific epigenetic lesions play a role by impacting set of genes important for malignant transformation. 

The alterations in DNA methylation landscape in pediatric AML emphasizes the use of epigenetic therapies in pediatric AML. Our results from a recent integrated analysis of methylation and RNA expression with minimal residual disease (MRD), event-free survival (EFS), and overall survival (OS) established that decreased methylation and increased RNA expression of DNMT3B associates with worse prognosis. Additionally, DNMT3B expression was correlated with greater genome-wide methylation burden which was associated with poor outcome. Our current results highlight cytogenetics-specific unique signatures that hold prognostic value and might in part contribute to the differences in outcome by cytogenetics. Further, complimentary epigenetic and genetic lesions point to certain vulnerable genomics hotspots of relevance in AML. In adults, DNA methyltransferase inhibitors (DNMTis) as azacytidine and decitabine have shown promising results when used in different combinations (recent review [73]). The differences in abundance of features used for risk group stratification of AML differ in pediatric and adult AML (e.g., higher abundance of DNMT3A mutations and lower abundance of t(8:21), inv(16) of 11q23 rearrangements in adults as compared to pediatric AML) has been well-established [74]. Our results highlight distinct methylation signatures within these cytogenetic categories in pediatrics, providing a rationale for a potential addition of epigenetic targeting therapy as DNMTis in pediatric AML. This additional layer of deregulated epigenetic signatures that co-occur complimentary to the chromosomal/mutations landscape suggests a possible synergistic interaction to potentiate leukemogenesis and a potential to be targeted by epigenetic therapy. Thus, based on our previous and current results from AML02 trial and evidence from adult AML showing potential benefit of DNMTis, the epigenetic priming using decitabine and azacytidine is being tested in the ongoing pediatric AML16 clinical trial (NCT00703820) with patients being enrolled across multiple centers in the US. Results from this trial and other ongoing studies will provide relevant information to guide epigenetic therapy in combination with other agents based on co-existing cytogenetic and epigenetic markers.

## 5. Conclusions

In summary, though the driver and passenger mutations that drive leukemogenesis in AML are still being defined, the genes impacted by genetic lesions or mutations have been mapped primarily to signaling molecules, transcription factors or epigenetic genes (e.g., *FLT3*, *Ras*, *AML1*, *C/EBPa*, *IDH1*, *IDH2*, *DNMT3A*, *PU.1*) and those effected by chromosomal rearrangements are primarily mapped to transcription factors of relevance in hematopoiesis (e.g., *PML-RARa*, *PLZF-RARa*, *CEBPa-MYH11*, *AML-ETO [RUNX1T1-RUNX1]*). Our results show another layer of deregulated epigenetic signatures that co-occur complimentary to the chromosomal/mutations landscape, suggesting a possible synergistic interaction to potentiate leukemogenesis. Epigenetic dysregulation of oncogenes and tumor suppressors that are subtype specific suggest malignant transformation probably leverages both genetic and epigenetic mechanisms to impact biological pathways critical for leukemogenesis.

## Figures and Tables

**Table 1:** Summary of methylation and Gene expression clusters identified by unsupervised analysis.

**Methylation Subgroup**	**11q23 (*n* = 23)**	**Insuff (*n* = 3)**	**inv(16) (*n* = 22)**	**Miscell (*n* = 47)**	**Normal (N = 39)**	**t(8;21) (*n* = 24)**	**t(9;11) (*n* = 14)**	**Total**	**MRD-Inevaluable (*n* = 12)**	**MRD-Negative *n* (%)**	**MRD-Positive *n* (%)**
A	0	0	20	3	1	1	0	25	0	22 (88%)	3 (12%)
B	16	1	0	17	7	0	13	54	7 (13%)	31 (57.4%)	16 (29.6%)
C	1	0	0	4	0	0	0	5	0	2 (40%)	3 (60%)
D	3	1	0	2	3	0	1	10	1 (10%)	3 (30%)	6 (60%)
E	3	1	1	11	26	0	0	42	3 (7.15%)	14 (33.3%)	25 (59.5%)
F	0	0	0	0	1	21	0	22	1 (4.5%)	16 (72.72%)	5 (22.7%)
O	0	0	1	10	1	2	0	14	0	5 (35.71%)	9 (64.3%)
**Expression Subgroup**	**11q23 (*n* = 21)**	**Insuff (*n* = 1)**	**inv(16) (*n* = 21)**	**Miscell (*n* = 45)**	**Normal (N = 42)**	**t(8;21) (*n* = 24)**	**t(9;11) (*n* = 11)**	**Total**	**MRD-Inevaluable (*n* = 12)**	**MRD-Negative *n* (%)**	**MRD-Positive *n* (%)**
0	2	0	1	9	6	1	1	20	0	9 (45%)	11 (55%)
1	8	0	18	19	16	0	1	62	2 (3.2%)	40 (64.5%)	20 (32.3%)
2	1	0	0	0	0	0	6	7	1 (14.3%)	6 (85.7%)	0 (0%)
3	3	0	0	2	12	0	0	17	2 (11.7%)	7 (41.2%)	8 (47.1%)
4	0	0	1	1	1	23	0	26	0	19 (73.1%)	7 (26.9%)
5	7	1	1	9	7	0	3	28	7 (38.8%)	10 (35.7%)	11 (39.3%)
6	0	0	0	5	0	0	0	5	0	0 (0%)	5 (100%)

**Table 2:** Top 50 methylation probes identified by supervised analysis of DNA methylation with to 50 unique methylation signatures by individual cytogenetic risk feature shown.

inv(16) vs. Non inv(16)				t(8;21) vs. Non t(8;21)				t(9;11) vs Non t(9;11)				Other 11q23 vs Non 11q23				Normal Cytogenetics vs. Non Normal Cytogentics				Other Abnormalities vs. Not			
illumina_ID	CHR	Gene Name	inv(16)vs. Non inv 16 *p* Value	illumina_ID	CHR	Gene Name	t(8;21) vs. Non t(8;21) *p* Value	illumina_ID	CHR	Gene Name	t(9;11) vs. Non t(9;11) *p* Value	illumina_ID	CHR	Gene Name	11q23 vs. Non 11q23 *p* Value	illumina_ID	CHR	Gene Name	Normal vs. non Normal *p* Value	illumina_ID	CHR	Gene Name	Others vs. Non Others *p* Value
cg11757040	14	DPF3	1.08 × 10^−19^	cg06640020	6	BX649158	1.10 × 10−29	cg27486692	12	PLBD1	1.07 × 10^−13^	cg22364219	1	AL832937	1.16 × 10^−10^	cg04498190	1	TRIM46	1.21 × 10^−10^	cg04738464	2	TNS1	6.66 × 10^−10^
cg13588403	14	DPF3	1.38 × 10^−19^	cg00948274	16	SOLH	1.07 × 10^−27^	cg25670451	12	SCNN1A	2.55 × 10^−13^	cg05817709	11	RARRES3	2.22 × 10^−9^	cg13372766	1	OBSCN	1.42 × 10^−10^	cg02147126	19	AZU1	8.94 × 10^−10^
cg01138706	2	TNS1	1.65 × 10^−17^	cg04194341	13	DZIP1	2.15 × 10^−27^	cg20373894	2	BC071802	7.20 × 10^−13^	cg18061433	10	C10orf113	6.98 × 10^−9^	cg13199615	1	TRIM46	2.43 × 10^−9^	cg26677004	1	TOR1AIP2	1.04 × 10^−9^
cg26953469	10	JA682651	2.88 × 10^−17^	cg09057885	21	COL18A1	2.15 × 10^−27^	cg15605307	11	MIR1237	9.99 × 10^−13^	cg07568841	7	MIR550B1	8.02 × 10^−9^	cg18832152	1	EFNA3	3.79 × 10^−9^	cg23678154	2	MIR3126	3.74 × 10^−9^
cg15256743	12	PLXNC1	7.04 × 10^−17^	cg24378253	1	RNF223	3.00 × 10^−27^	cg01219000	4	CXCL5	1.70 × 10^−12^	cg04753583	21	PCBP3	8.02 × 10^−9^	cg15474859	2	KLF7	1.80 × 10^−9^	cg25361961	19	ZNF550	3.83 × 10^−9^
cg26909217	16	ZFHX3	1.31 × 10^−15^	cg00502209	15	LRRK1	4.12 × 10^−27^	cg19954448	17	CYTH1	1.88 × 10^−12^	cg13933080	10	SPAG6	5.27 × 10^−8^	cg06500792	2	AK097952	2.56 × 10^−9^	cg01877450	7	BRI3	7.62 × 10^−9^
cg09221269	21	RUNX1	1.31 × 10^−15^	cg07088935	2	TPO	1.01 × 10^−26^	cg00722188	14	CCDC88C	2.55 × 10^−12^	cg03124514	5	AK056817	6.16 × 10^−8^	cg02913882	3	TBL1XR1	1.80 × 10^−9^	cg15134583	1	TP53BP2	1.07 × 10^−8^
cg15796340	17	PRCD	1.42 × 10^−15^	cg24216893	2	TPO	1.01 × 10^−26^	cg00704819	6	ANKRD6	2.82 × 10^−12^	cg01067139	5	AK056817	1.11 × 10^−7^	cg05429527	6	FOXC1	5.86 × 10^−10^	cg15487251	3	IGF2BP2	1.12 × 10^−8^
cg09599228	4	RXFP1	1.91 × 10^−15^	cg02914962	6	BX649158	1.34 × 10^−26^	cg19690404	7	CPA4	4.18 × 10^−12^	cg13494087	9	KLF4	2.33 × 10^−7^	cg25583983	6	MICA	9.57 × 10^−10^	cg18780288	10	XPNPEP1	1.25 × 10^−8^
cg06343673	17	RPTOR	3.43 × 10^−15^	cg04734276	5	AX747985	1.76 × 10^−26^	cg03969651	17	TBX4	4.60 × 10^−12^	cg01146808	6	PRDM1	4.77 × 10^−7^	cg27144224	6	MICA	1.06 × 10^−9^	cg02119938	15	ACSBG1	1.34 × 10^−8^
cg19755069	1	CSF1	4.26 × 10^−15^	cg05408203	16	CACNA1H	3.87 × 10^−26^	cg07877629	16	CMIP	6.12 × 10^−12^	cg19744908	1	LPHN2	6.85 × 10^−7^	cg20028974	6	SLC2A12	1.27 × 10^−9^	cg10752406	19	AZU1	1.53 × 10^−8^
cg20536921	15	MEF2A	5.65 × 10^−15^	cg05019221	2	OTOS	8.10 × 10^−26^	cg22042908	20	RASSF2	7.38 × 10^−12^	cg08004620	8	AX747124	1.31 × 10^−6^	cg12837744	6	SYNGAP1	1.48 × 10^−9^	cg14870461	3	C3orf64	1.94 × 10^−8^
cg20331980	6	TAB2	6.98 × 10^−15^	cg04011470	8	PHYHIP	8.10 × 10^−26^	cg10100652	10	LOC643529	8.88 × 10^−12^	cg19743406	6	LHFPL5	1.54 × 10^−6^	cg24899846	6	MICA	3.61 × 10^−9^	cg16535752	17	MIR4729	2.03 × 10^−8^
cg23013977	19	CLIP3	9.20 × 10^−15^	cg13967936	1	GJA4	1.29 × 10^−25^	cg15063726	11	HPX	1.06 × 10^−11^	cg08390877	2	EN1	1.67 × 10^−6^	cg05852760	7	IGF2BP3	4.51 × 10^−10^	cg04087271	1	CDA	2.36 × 10^−8^
cg27587645	19	BTBD2	1.39 × 10^−14^	cg12946705	6	FAM120B	1.29 × 10^−25^	cg11726288	1	NPPA-AS1	1.16 × 10^−11^	cg01369082	5	BC045578	2.28 × 10^−6^	cg19507725	7	HIP1	5.28 × 10^−10^	cg16643542	19	AZU1	2.42 × 10^−8^
cg08124030	3	TM4SF1	2.36 × 10^−14^	cg13751417	9	VAV2	2.54 × 10^−25^	cg13412283	7	AK024243	1.27 × 10^−11^	cg25714774	2	TRAPPC12	2.34 × 10^−6^	cg10902549	10	MTG1	3.35 × 10^−9^	cg07018107	8	MIR1208	2.94 × 10^−8^
cg20299572	11	MOB2	2.69 × 10^−14^	cg08178815	20	ADRM1	3.15 × 10^−25^	cg04241282	3	LARS2	1.39 × 10^−11^	cg07217350	5	MAT2B	2.34 × 10^−6^	cg18571531	11	AK097446	6.17 × 10^−10^	cg03082779	20	LOC100131496	2.94 × 10^−8^
cg22549879	2	PKI55	3.72 × 10^−14^	cg17712092	4	LARP1B	3.91 × 10^−25^	cg11235411	10	FW312330	1.39 × 10^−11^	cg11702085	6	TRNA_Leu	2.34 × 10^−6^	cg00667026	14	ZFP36L1	3.20 × 10^−10^	cg23606421	9	ENTPD8	3.07 × 10^−8^
cg04596071	7	MAD1L1	4.51 × 10^−14^	cg05716567	8	ZC3H3	3.91 × 10^−25^	cg09107053	1	C1orf198	1.66 × 10^−11^	cg01826444	1	FNDC7	2.40 × 10^−6^	cg14876117	14	PYGL	2.62 × 10^−9^	cg14663914	19	AZU1	3.20 × 10^−8^
cg02065790	12	PPFIBP1	6.19 × 10^−14^	cg13593887	9	C9orf62	3.91 × 10^−25^	cg15889057	12	ITGB7	1.66 × 10^−11^	cg06626359	14	PRIMA1	2.46 × 10^−6^	cg15698066	14	EVL	3.19 × 10^−9^	cg13551117	2	Mir_562	3.27 × 10^−8^
cg11944797	13	STK24	1.22 × 10^−13^	cg07912453	15	PGPEP1L	4.82 × 10^−25^	cg05000598	1	TTC24	1.81 × 10^−11^	cg18281743	5	STC2	2.73 × 10^−6^	cg07199524	15	CRTC3	3.37 × 10^−10^	cg10221596	2	ALS2CR12	3.34 × 10^−8^
cg10839684	17	LOC100499466	1.55 × 10^−13^	cg27111634	9	DQ593554	5.93 × 10^−25^	cg09579869	1	Mir_584	1.97 × 10^−11^	cg20312179	1	EPS15	3.10 × 10^−6^	cg18942110	15	CRTC3	9.82 × 10^−10^	cg00153306	3	TMIE	3.34 × 10^−8^
cg03451731	17	GP1BA	1.65 × 10^−13^	cg23731501	1	MIR429	7.28 × 10^−25^	cg21642076	22	TMEM191A	1.97 × 10^−11^	cg03847796	1	MAB21L3	3.10 × 10^−6^	cg19134665	15	CRTC3	1.76 × 10^−9^	cg03216580	10	KLF6	3.34 × 10^−8^
cg05247914	19	FXYD1	1.65 × 10^−13^	cg08166720	17	5S_rRNA	1.09 × 10^−24^	cg17262492	20	PCIF1	2.34 × 10^−11^	cg01296593	16	FOXC2	3.51 × 10^−6^	cg27326306	16	AK056683	1.99 × 10^−9^	cg16808455	18	TUBB6	4.05 × 10^−8^
cg16747164	4	TLR10	3.16 × 10^−13^	cg13028635	11	LSP1	1.32 × 10^−24^	cg12167489	8	RBPMS	2.55 × 10^−11^	cg12354192	3	Y_RNA	3.60 × 10^−6^	cg18200326	16	FHOD1	2.43 × 10^−9^	cg24534743	1	AHDC1	4.14 × 10^−8^
cg17384769	6	LOC100128176	3.76 × 10^−13^	cg17820878	1	SLC9A1	1.61 × 10^−24^	cg09518969	11	PLA2G16	2.55 × 10^−11^	cg06626126	3	MECOM	3.79 × 10^−6^	cg24210813	17	HOXB5	5.71 × 10^−14^	cg16727006	16	ZCCHC14	4.14 × 10^−8^
cg18908677	10	BAG3	3.98 × 10^−13^	cg17696563	2	NEU4	1.61 × 10^−24^	cg14949065	2	TCF23	2.78 × 10^−11^	cg20961007	1	AX747534	3.88 × 10^−6^	cg05487507	17	HOXB5	1.55 × 10^−12^	cg09053680	10	UTF1	4.80 × 10^−8^
cg08715862	16	FHOD1	4.21 × 10^−13^	cg26786253	17	5S_rRNA	1.94 × 10^−24^	cg05498785	16	IRF8	2.78 × 10^−11^	cg12610471	10	SPAG6	3.88 × 10^−6^	cg12744859	17	HOXB5	3.08 × 10^−12^	cg13521941	9	ENTPD8	4.90 × 10^−8^
cg03440386	7	TCRBV9S1A1T	5.60 × 10^−13^	cg18851522	14	KIF26A	2.35 × 10^−24^	cg22718696	1	MTOR	3.02 × 10^−11^	cg07777156	17	TRNA	3.88 × 10^−6^	cg07676709	17	HOXB6	4.06 × 10^−12^	cg23098529	19	PPAN-P2RY11	5.12 × 10^−8^
cg10368834	16	RUNDC2C	6.63 × 10^−13^	cg01074955	1	NPHP4	3.40 × 10^−24^	cg05483534	2	BC048424	3.02 × 10^−11^	cg23361764	11	ODZ4	4.08 × 10^−6^	cg18127922	17	HOXB6	8.14 × 10^−12^	cg04848343	19	PLIN5	5.22 × 10^−8^
cg14381313	16	AK126852	7.42 × 10^−13^	cg14141549	7	DQ583756	3.40 × 10^−24^	cg01391022	12	WDR66	3.28 × 10^−11^	cg23739746	13	RASA3	4.62 × 10^−6^	cg00711072	17	HOXB6	9.73 × 10^−12^	cg08498533	10	TTC40	5.57 × 10^−8^
cg20436086	10	COL13A1	7.84 × 10^−13^	cg06782041	1	LDLRAD2	4.08 × 10^−24^	cg06099459	12	C12orf77	3.28 × 10^−11^	cg02273477	17	NXN	4.62 × 10^−6^	cg00072689	17	HOXB6	1.00 × 10^−11^	cg16672770	16	PIEZO1	5.93 × 10^−8^
cg02035018	4	MXD4	8.29 × 10^−13^	cg08447200	22	TRABD	4.89 × 10^−24^	cg00979704	6	TNXB	3.56 × 10^−11^	cg07038400	3	PPP2R3A	5.49 × 10^−6^	cg24173049	17	SLC16A13	3.32 × 10^−11^	cg16378063	17	FLJ40194	6.05 × 10^−8^
cg17873456	13	EFNB2	9.78 × 10^−13^	cg23841288	7	AX746826	5.84 × 10^−24^	cg02746781	16	DPEP2	3.56 × 10^−11^	cg22429199	3	CRYBG3	5.77 × 10^−6^	cg26916621	17	MIR10A	4.41 × 10^−11^	cg17645677	5	PPARGC1B	6.58 × 10^−8^
cg00303450	2	MFSD6	1.03 × 10^−12^	cg22141235	11	MRGPRE	5.84 × 10^−24^	cg03543120	5	MIR143	3.87 × 10^−11^	cg00446123	20	LIME1	5.77 × 10^−6^	cg01405107	17	HOXB5	5.54 × 10^−11^	cg13984746	17	HEXDC	6.58 × 10^−8^
cg01541443	7	C7orf41	1.09 × 10^−12^	cg21876283	1	LDLRAD2	6.97 × 10^−24^	cg00468395	16	XPO6	3.87 × 10^−11^	cg07989568	17	TEKT1	5.91 × 10^−6^	cg10308785	17	HOXB6	6.37 × 10^−11^	cg01980222	6	TREM2	6.86 × 10^−8^
cg24400630	1	GBP5	1.15 × 10^−12^	cg13411506	13	MCF2L	8.30 × 10^−24^	cg03721994	19	MED16	3.87 × 10^−11^	cg23570923	4	HTRA3	6.21 × 10^−6^	cg00690402	17	HOXB5	8.19 × 10^−11^	cg07170045	8	FGF17	7.94 × 10^−8^
cg00346376	5	SLC12A7	1.15 × 10^−12^	cg01403811	22	ELFN2	8.30 × 10^−24^	cg14839919	7	C7orf45	4.20 × 10^−11^	cg16419354	1	FAM163A	6.36 × 10^−6^	cg04196862	17	LOC404266	1.87 × 10^−10^	cg07714276	6	RREB1	8.81 × 10^−8^
cg00059737	1	VPS13D	1.22 × 10^−12^	cg00450617	2	TPO	9.86 × 10^−24^	cg26164773	10	TIAL1	4.56 × 10^−11^	cg06179127	7	AK024243	6.36 × 10^−6^	cg01572694	17	MIR10A	2.38 × 10^−10^	cg06495615	19	ARID3A	8.81 × 10^−8^
cg11216632	1	ARHGEF2	1.29 × 10^−12^	cg16520049	17	FBF1	9.86 × 10^−24^	cg01826337	1	CALML6	4.94 × 10^−11^	cg09785391	18	ZFP161	6.36 × 10^−6^	cg21864868	17	LOC404266	2.87 × 10^−10^	cg04891053	1	PVRL4	8.99 × 10^−8^
cg21042919	17	ARHGAP23	1.43 × 10^−12^	cg02664177	7	PDGFA	1.17 × 10^−23^	cg11493223	17	TMC6	5.35 × 10^−11^	cg04392469	1	MOSC2	6.68 × 10^−6^	cg15435170	17	HOXB6	2.95 × 10^−10^	cg08190450	17	NXN	8.99 × 10^−8^
cg15031685	7	ATP6V1F	1.51 × 10^−12^	cg02113067	11	FERMT3	1.17 × 10^−23^	cg05165250	18	RAB31	5.35 × 10^−11^	cg22874893	6	RBM24	7.35 × 10^−6^	cg21816532	17	HOXB6	5.01 × 10^−10^	cg10973881	1	TMOD4	9.18 × 10^−8^
cg21950720	1	EXTL1	1.68 × 10^−12^	cg00155679	7	CNPY1	1.64 × 10^−23^	cg24166628	3	TNNC1	5.80 × 10^−11^	cg03930153	3	TBL1XR1	7.53 × 10^−6^	cg14232289	17	SSTR2	1.14 × 10^−9^	cg23327851	14	RNASE2	9.97 × 10^−8^
cg19939077	10	PPIF	1.68 × 10^−12^	cg15769475	17		1.64 × 10^−23^	cg05480883	10	ITIH5	5.80 × 10^−11^	cg16536330	8	LEPROTL1	7.53 × 10^−6^	cg01986016	17	HOXB6	1.99 × 10^−9^	cg12136387	12	MIR4472-2	1.04 × 10^−7^
cg15482928	1	PTPRF	1.98 × 10^−12^	cg05345823	11	MUC6	1.93 × 10^−23^	cg12146447	17	TAC4	5.80 × 10^−11^	cg16949914	10	IL2RA	8.09 × 10^−6^	cg01723934	17	HOXB6	2.26 × 10^−9^	cg17125472	11	AK094674	1.06 × 10^−7^
cg04155862	3	MGLL	1.98 × 10^−12^	cg05322931	17	NXN	2.27 × 10^−23^	cg15064964	2	ASB1	7.35 × 10^−11^	cg18075627	12	MIR1178	8.29 × 10^−6^	cg22660299	17	LOC404266	2.89 × 10^−9^	cg06383124	16	LOC400548	1.15 × 10^−7^
cg11667400	13	UBAC2	2.09 × 10^−12^	cg21268658	1	PTPRF	2.67 × 10^−23^	cg22014983	2	U6	7.35 × 10^−11^	cg15871647	4	PARM1	8.49 × 10^−6^	cg10749413	19	ABCA7	2.42 × 10^−11^	cg23230830	1	LOC646627	1.17 × 10^−7^
cg03790192	16	BANP	2.09 × 10^−12^	cg02960418	7	PDGFA	3.14 × 10^−23^	cg22870280	15	BCL2L10	7.94 × 10^−11^	cg02603128	9	FRMD3	8.49 × 10^−6^	cg01614597	19	LOC113230	3.35 × 10^−9^	cg08493063	16	RMI2	1.22 × 10^−7^
cg05497253	5	LHFPL2	2.32 × 10^−12^	cg07503662	16	KLHDC4	3.14 × 10^−23^	cg05923736	10	ECHS1	9.27 × 10^−11^	cg24891133	13	C13orf33	8.91 × 10^−6^	cg21053323	21	SUMO3	3.11 × 10^−10^	cg19573490	17	PCYT2	1.30 × 10^−7^
cg14749678	3	CCRL2	2.72 × 10^−12^	cg03614513	1	TSPAN2	4.31 × 10^−23^	cg26616258	16	PDIA2	1.00 × 10^−10^	cg09335658	7	RBM28	9.12 × 10^−6^	cg16932065	22	LIMK2	2.82 × 10^−9^	cg18734433	7	ZNF775	1.33 × 10^−7^

Black Font: Genes hypomethylated in the selected cytogenetic group as compared to other. Red Font: Genes hypermethylated in the selected cytogenetic group as compared to other.

**Table 3:** Complementary methylation deregulation of AML hot spot genomic regions by cytogenetic characteristics.

Cytogenetic Subgroup	Chr	Gene	GroupMedian: Presence of Cytogenetic Feature	GroupMedian: Absence of Cytogenetic Feature	*p* Value	FDR Adjusted *p*	Complementary Cytogenetic Lesion
inv(16) [CBFB-MYH11]	16	*CBFB*	2.456	1.012	3.97 × 10^−14^	3.08 × 10^−10^	Cis-cytogenetic lesion
inv(16) [CBFB-MYH11]	16	*MYH11*	2.448	4.063	3.18 × 10^−12^	7.26 × 10^−9^	Cis-cytogenetic lesion
inv(16) [CBFB-MYH11]	21	*RUNX1*	2.512	4.213	1.31 × 10^−15^	2.91 × 10^−11^	Trans Cytogenetic lesion-Fusion gene for t(8;21)
inv(16) [CBFB-MYH11]	01	*RUNX3*	−0.6088	0.7442	6.27 × 10^−13^	2.30 × 10^−9^	
inv(16) [CBFB-MYH11]	21	*RUNX1*	0.703	2.305	2.01 × 10^−11^	2.89 × 10^−8^	Trans Cytogenetic lesion-Fusion gene for t(8;21)
inv(16) [CBFB-MYH11]	21	*RUNX1*	−0.6719	0.9548	2.10 × 10^−11^	2.94 × 10^−8^	Trans Cytogenetic lesion-Fusion gene for t(8;21)
inv(16) [CBFB-MYH11]	1	*RUNX3*	−3.508	−0.8562	1.09 × 10^−10^	1.00 × 10^−7^	
inv(16) [CBFB-MYH11]	19	*FLT3LG*	−2.486	−0.4993	4.45 × 10^−11^	5.19 × 10^−8^	
inv(16) [CBFB-MYH11]	1	*GLIS1*	0.096	2.2	4.33 × 10^−12^	9.24 × 10^−9^	
Normal	17	*MLLT6*	0.348	−1.732	9.67 × 10^−9^	8.17 × 10^−6^	
O11q23 [MLL-X]	16	*CBFA2T3*	1.023	−1.555	4.58 × 10^−9^	4.92 × 10^−5^	Trans Cytogenetic lesion-Fusion gene inv16-CBFA2T3-GLIS2)
O11q23 [MLL-X]	16	*CBFA2T3*	2.398	0.9998	7.49 × 10^−9^	6.22 × 10^−5^	Trans Cytogenetic lesion-Fusion gene inv16-CBFA2T3-GLIS2)
O11q23 [MLL-X]	16	*CBFA2T3*	−0.2261	−2.417	3.71 × 10^−8^	0.00014808	Trans Cytogenetic lesion-Fusion gene inv16-CBFA2T3-GLIS2)
O11q23 [MLL-X]	16	*CBFA2T3*	1.76	−0.2214	6.36 × 10^−8^	0.00019022	Trans Cytogenetic lesion-Fusion gene inv16-CBFA2T3-GLIS2)
O11q23 [MLL-X]	16	*CBFA2T3*	−0.4395	−2.155	3.03 × 10^−7^	0.00039603	Trans Cytogenetic lesion-Fusion gene inv16-CBFA2T3-GLIS2)
O11q23 [MLL-X]	09	*MLLT3*	−0.6595	−1.311	3.59 × 10^−8^	0.00014478	
t(8;21) [RUNX1-RUNX1T1]	16	*CBFA2T3*	−0.5581	3.259	1.93 × 10^−23^	6.22 × 10^−20^	Trans Cytogenetic lesion-Fusion gene inv16-CBFA2T3-GLIS2)
t(8;21) [RUNX1-RUNX1T1]	16	*CBFA2T3*	0.1236	3.28	2.97 × 10^−22^	5.79 × 10^−19^	Trans Cytogenetic lesion-Fusion gene inv16-CBFA2T3-GLIS2)
t(8;21) [RUNX1-RUNX1T1]	16	*CBFA2T3*	0.0312	−3.232	5.93 × 10^−22^	1.04 × 10^−18^	Trans Cytogenetic lesion-Fusion gene inv16-CBFA2T3-GLIS2)
t(8;21) [RUNX1-RUNX1T1]	16	*CBFA2T3*	0.1551	2.453	2.96 × 10^−20^	2.57 × 10^−17^	Trans Cytogenetic lesion-Fusion gene inv16-CBFA2T3-GLIS2)
t(8;21) [RUNX1-RUNX1T1]	16	*CBFA2T3*	0.1614	3.657	6.32 × 10^−20^	4.85 × 10^−17^	Trans Cytogenetic lesion-Fusion gene inv16-CBFA2T3-GLIS2)
t(8;21) [RUNX1-RUNX1T1]	06	*MLLT4*	−0.1954	3.923	1.10 × 10^−29^	1.30 × 10^−24^	Trans Cytogenetic lesion MLL fusion partner (11q23)
t(8;21) [RUNX1-RUNX1T1]	19	*MLLT1*	−1.907	2.869	7.39 × 10^−28^	3.74 × 10^−23^	Trans Cytogenetic lesion MLL fusion partner (11q23)
t(8;21) [RUNX1-RUNX1T1]	6	*MLLT4*	0.4282	5.277	1.34 × 10^−26^	2.15 × 10^−22^	Trans Cytogenetic lesion MLL fusion partner (11q23)
t(8;21) [RUNX1-RUNX1T1]	6	*MLLT4*	−2.402	2.485	6.86 × 10^−23^	1.80 × 10^−19^	Trans Cytogenetic lesion MLL fusion partner (11q23)
t(9;11)	22	*MYH9*	1.131	2.282	1.17 × 10^−10^	9.12 × 10^−8^	Trans Cytogenetic lesion MLL fusion partner (11q23)

## Supplementary Materials

The following are available online at https://www.mdpi.com/2072-6694/12/10/3024/s1, Figure S1: The bootstrap mean Dunn Index and bootstrap reproducibility of 793 methylation HCMs, Figure S2: A heatmap of cytogenetics and subgroup assignments of the nine candidate HCMs, Figure S3: The bootstrap mean Dunn Index and bootstrap reproducibility of expression HCMs, Figure S4: Subgroup assignments of the candidate expression HCMs, Table S1: Patient Characteristics, Table S2: Five hundred CpG sites that defined 7 methylation groups in an unsupervised bootstrap clustering analysis in AML patients, Table S3: Seven hundred probes that defined 7 expression groups in an unsupervised bootstrap clustering analysis in AML patients, Table S4: Paired methylation-expression supervised analysis of top 233 pairs along with summary of the cytogenetic features, Table S5: Top 100 Methylation signatures differentiating FLT3-ITD with noj FLT3-ITD cases.
