# Supplementary material for: DNA Methylation Clusters and Their Relation to Cytogenetic Features in Pediatric AML
Source: Cancers (Basel). 2020 Oct 17;12(10):3024. doi: 10.3390/cancers12103024 (PMC7603219; doi:10.3390/cancers12103024)
Supplement: Supplementary file 1 [file cancers-12-03024-s001.zip › cancers-962544-Supplementary/cancers-962544-Supplementary .pdf]

Supplementary Materials

DNA Methylation Clusters and Their Relation to Cytogenetic Features in Pediatric AML

Jatinder K Lamba, Xueyuan Cao, Susana Raimondi, James Downing, Raul Ribeiro, Tanja A. Gruber, Jeffrey Rubnitz and Stanley Pounds

Supplementary Material

Supplementary Method1: Methylation Cluster Analysis

We used a bootstrap procedure to evaluate the reproducibility and distinctiveness (Dunn Index) of 4032 hierarchical clustering methods (HCMs) that used 8 criteria to select m = 1, 2, 3, …, 9, 10, 20, 30, …, 100, 200, 300, …, 1000 probe-sets to define k = 2, 3, 4, …, 10 subgroups by average or complete linkage (4032 HCMs = 8 possible criteria × 28 possible numbers of features × 9 possible number of subgroups × 2 linkage methods). Each HCM was applied to the observed data set and a series of 500 bootstrap data sets obtained by resampling subjects with replacement. For each HCM, the label of each cluster in each bootstrap iteration was mapped back to the most common label of those subjects in the observed data. In this way, each HCM had the clusters labeled in the same way in the observed data and across all bootstraps. Then, for each HCM, the *best assignment probability* of each individual was the frequency of the most common cluster label across all bootstrap data sets. For each HCM, the bootstrap aggregated (bagged) cluster label was the most common cluster label across all bootstrap data sets. Then, the *bootstrap reproducibility* of the HCM was computed as the mean of the best assignment probability over all individuals. Also, for each HCM, the Dunn Index was computed for the cluster results in each bootstrap iteration. For each HCM, the bootstrap mean Dunn Index was the mean of the Dunn Index over all bootstrap iterations. In this way, the bootstrap procedure quantified the stability of each HCM with the bootstrap reproducibility and the distinctiveness of each HCM’s cluster assignments with the bootstrap mean Dunn Index.

The results of the bootstrap were used to select candidate HCMs for further consideration. HCMs that failed to recapitulate the number of requested clusters k in the bagging cluster labels were eliminated from consideration. Also, HCMs that sought to use fewer than 2k features to define k clusters were also eliminated from consideration. For methylation clustering, 793 HCMs successfully recapitulated the specified number of clusters k in the bagged cluster labels and used at least 2k features to define k clusters. Figure S1 shows the bootstrap reproducibility and bootstrap mean Dunn Index of these HCMs. The product of the proportion of subjects assigned to a cluster (i.e. not declared outliers), bootstrap reproducibility, and bootstrap mean Dunn Index was used as an index to prioritize consideration of the remaining HCMs. For each number of clusters k, the HCM with the greatest product of the bootstrap mean Dunn Index and bootstrap reproducibility was considered as a candidate HCM. This gave a set of 9 candidate HCMs.

HCM 906, which used the 500 probe-sets with greatest value of the Hartigan and Hartigan dip statistic (1985) for bimodality to define 7 subgroups with complete linkage, was chosen as the final result of the methylation subgroup analysis. As shown in Figure S2, HCMs 3529, 857, 858, 868, and 482 defined 2–6 subgroups but failed to distinguish between the well-known t(8;21), inv(16), and MLL-rearranged AML subgroups. HCM 906 clearly distinguished between these three well-known subgroups and defined four additional subgroups. HCMs 2509, 2472, and 2511 failed to clearly identify those groups and had noticeably worse bootstrap reproducibility and/or mean bootstrap Dunn Index.

The analysis results clearly show that many probe-sets have a bimodal methylation that define reproducible and distinct subgroups that align closely with cytogenetic subgroups. There are other probe-sets that have great variation in methylation that define other kinds of subgroups that are reproducible and distinct.

Supplementary Method2: Expression Clustering

Clustering of microarray gene expression clustering was performed in a similar way. The same bootstrap procedure was used to evaluate the reproducibility and distinctiveness of subgroups produced by 4032 HCMs. A total of 1047 HCMs satisfied the criteria to be considered as candidates and nine candidate HCMs that defined 2–10 subgroups were chosen for further consideration (Figure S3). Among these 1047 HCMs, the nine HCMs that had the greatest product of the bootstrap reproducibility and mean bootstrap Dunn Index for their given number of clusters were chosen for further consideration. The subgroup assignments of these 9 HCMs are shown in Figure S4. Among these 9 HCMs, HCM 2463 was chosen as result to report. HCM 2463 used the 700 probe-sets with greatest median absolute deviation to define 7 subgroups by average linkage. HCM 2463 had similar bootstrap mean Dunn Index as the other eight candidate HCMs, was the only candidate HCM to delineate two major cytogenetic subgroups, and was one of 6 candidate HCMs to have better than 70% bootstrap reproducibility.

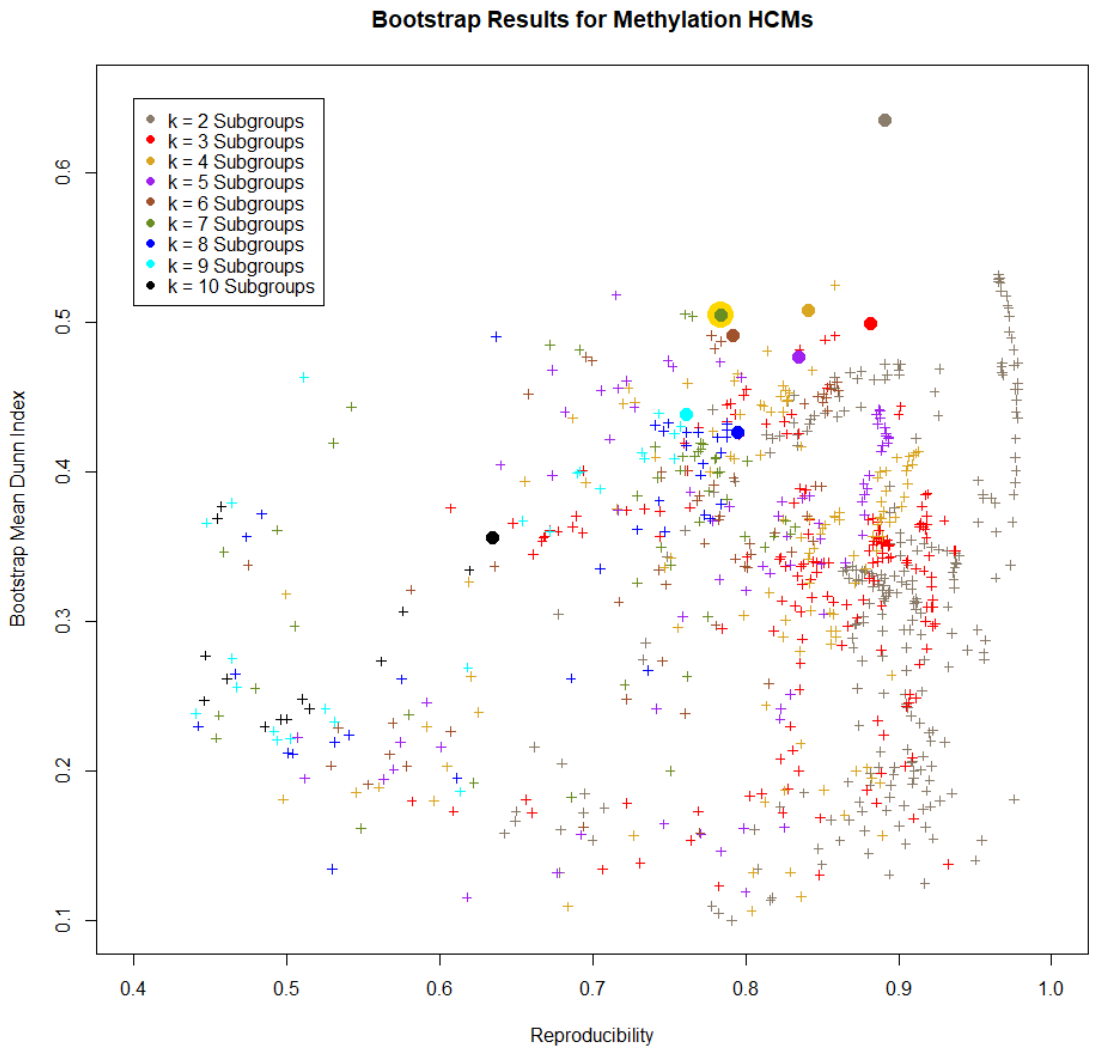

Figure S1. The bootstrap mean Dunn Index and bootstrap reproducibility of 793 methylation HCMs that satisfied criteria for further consideration (shown as crosses), and the 9 HCMs that had the greatest product of mean bootstrap Dunn Index and bootstrap reproducibility for a given number of clusters k (shown as large filled in circles). The number of clusters to be defined by each HCM are indicated by the color scheme in the top-left legend. The green circle highlighted by yellow boundary represents methylation HCM 906, which used the 500 methylation probes with greatest dip statistic to define 7 methylation clusters.

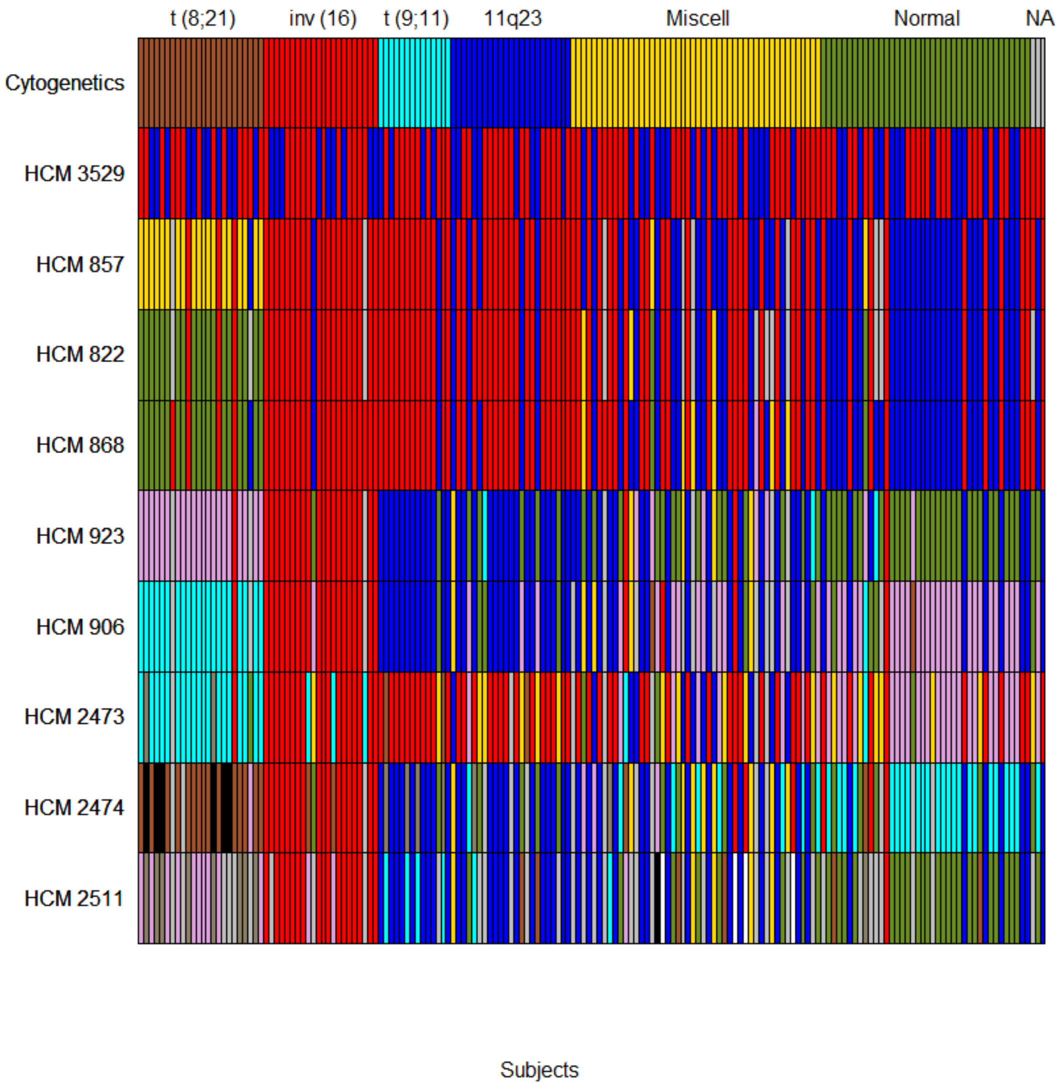

Figure S2. A heatmap of cytogenetics and subgroup assignments of the nine candidate HCMs. Each column represents a subject and each row represents either the cytogenetic subgroup (row 1) or the subgroups defined by a candidate HCM (subsequent rows).

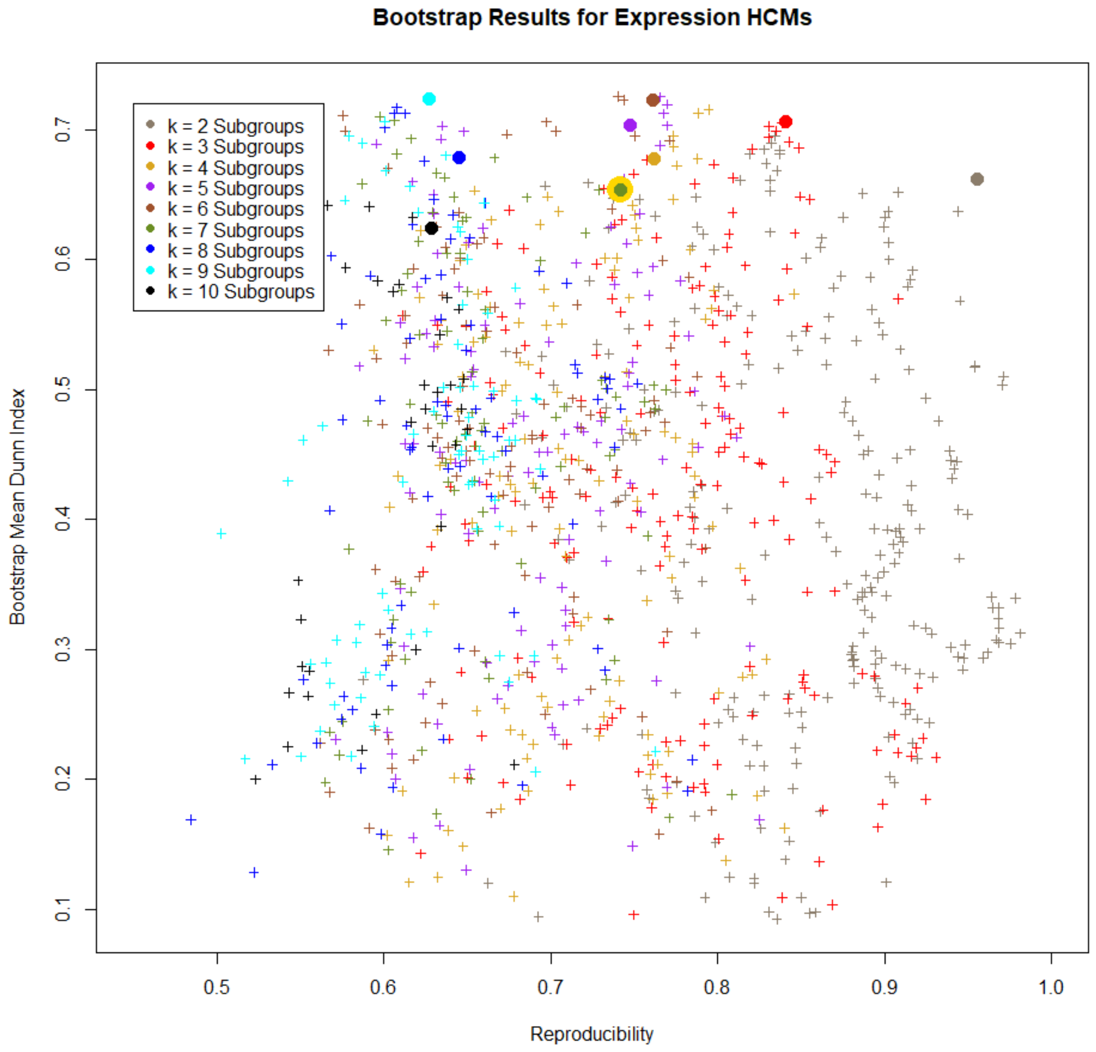

Figure S3. The bootstrap mean Dunn Index and bootstrap reproducibility of expression HCMs that satisfied criteria for further consideration (shown as crosses), and the 9 HCMs that had the greatest product of mean bootstrap Dunn Index and bootstrap reproducibility for a given number of clusters k (shown as large filled in circles). The number of clusters to be defined by each HCM are indicated by the color scheme in the top-left legend. The green circle highlighted by yellow boundary represents expression HCM 2463, which used the 700 probe-sets with greatest median absolute deviation to define 7 subgroups.

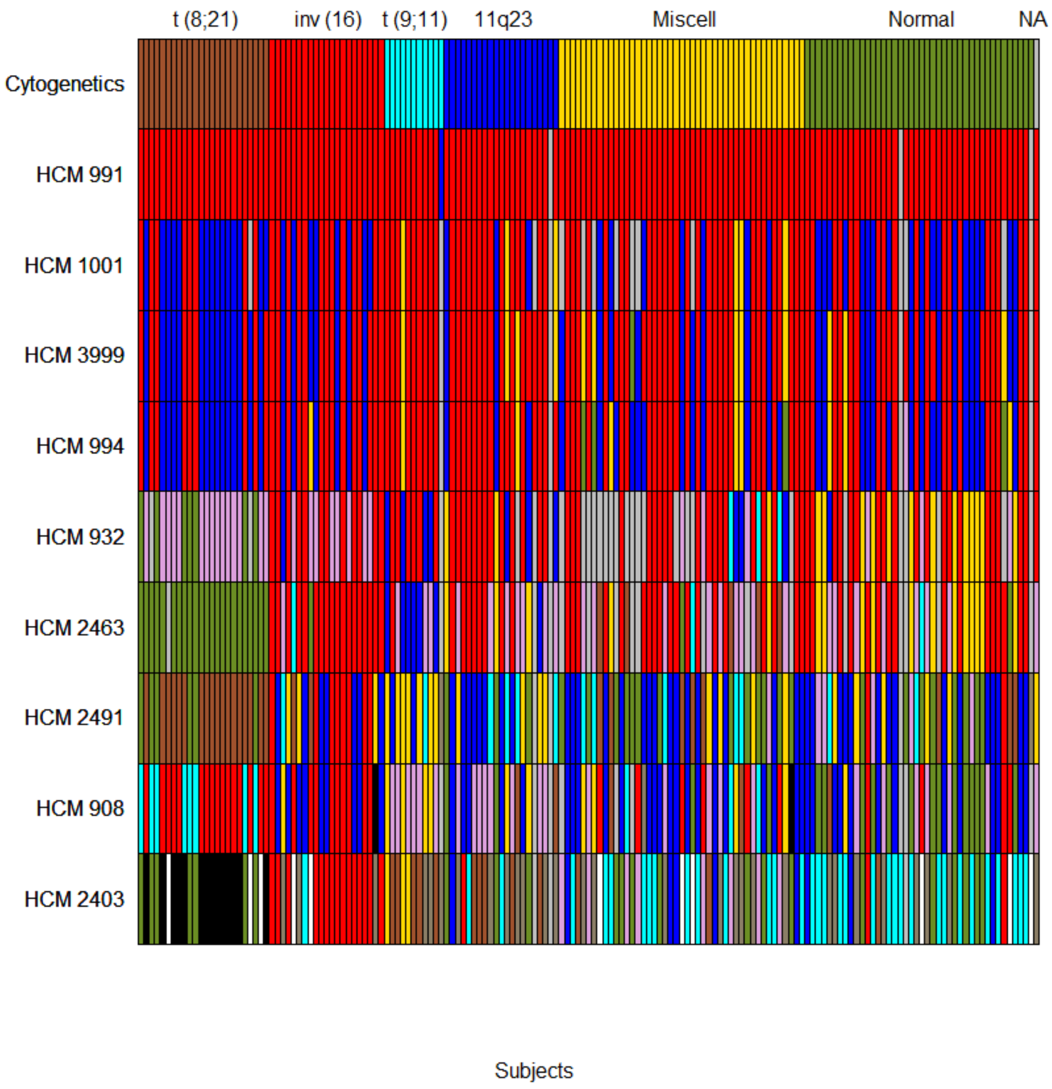

Figure S4. Subgroup assignments of the nine candidate expression HCMs.

Supplementary References

1. Hartigan, P.M. Computation of the Dip Statistic to Test for unimodality. *Appl. Stat. (JRSS C)* **1985**, *34*, 320–325.
2. Dunn, J. Well separated clusters and optimal fuzzy partitions. *J. Cybern.* **1974**, *4*, 95–104.

© 2020 by the authors. Licensee MDPI, Basel, Switzerland. This article is an open access article distributed under the terms and conditions of the Creative Commons Attribution (CC BY) license (http://creativecommons.org/licenses/by/4.0/).
